# Knowledge mapping of the links between the microbiota and allergic diseases: A bibliometric analysis (2002–2021)

**DOI:** 10.3389/fimmu.2022.1045795

**Published:** 2022-10-28

**Authors:** Hao Lv, Yunfei Wang, Ziang Gao, Peiqiang Liu, Danxue Qin, Qingquan Hua, Yu Xu

**Affiliations:** ^1^ Department of Otolaryngology-Head and Neck Surgery, Renmin Hospital of Wuhan University, Wuhan, China; ^2^ Research Institute of Otolaryngology-Head and Neck Surgery, Renmin Hospital of Wuhan University, Wuhan, China

**Keywords:** microbiota, allergic diseases, developing trends, bibliometric analysis, visual analysis

## Abstract

**Background:**

In recent decades, dramatic changes in modern environmental exposures and lifestyles have resulted in a steep rise in the prevalence of allergic diseases such as asthma, allergic rhinitis, atopic dermatitis and food allergies. Evidence is mounting that the microbiota plays a crucial role in allergic disorder development and evolution. Therefore, a better understanding of allergic diseases within the context of the microbiota is urgently needed. This work aimed to comprehensively outline general characteristics, research hotspots, evolution routes, and emerging trends in this area.

**Methods:**

Relevant publications from January 2002 to December 2021 were obtained from the Web of Science Core Collection on 5 August 2022. Bibliometric and visual analyses were performed using CiteSpace; VOSviewer; an online bibliometric platform; and Microsoft Excel 2019.

**Results:**

In total, 2535 documents met the requirements. The annual number of publications has shown rapid growth in the last two decades. The USA, University of California System, and Isolauri E of the University of Turku were the most productive and influential country, institution, and author, respectively. The *Journal of Allergy and Clinical Immunology* was the most prolific and most cocited journal. High-frequency keywords included “gut microbiota”, “asthma”, “atopic dermatitis”, “children”, and “probiotics”. Recent studies have focused on “atopic dermatitis”, “skin”, “asthma”, and “probiotics”, according to the cocitation analysis of references. Burst detection analysis of keywords showed that “community”, “skin microbiome”, “microbiome”, “*Staphylococcus aureus*”, and “chain fatty acid” were emerging research frontiers, which currently have ongoing bursts.

**Conclusion:**

In the last 20 years, studies of the microbiota in allergic diseases have been flourishing, and the themes have been increasing in depth. These findings provide valuable references on the current research hotspots and gaps and development trends in the link between the microbiota and allergic diseases.

## Introduction

Allergic diseases represent a heterogeneous group of disorders, including asthma, allergic rhinitis (AR), atopic dermatitis (AD), and food allergy (FA), characterized by dominant type 2 immune responses and IgE responses (1). It is well known that these allergic diseases share a strong epidemiologic and pathophysiological association (2, 3). For example, Celakovska et al. (4) reported that AD patients with confirmed FA are significantly more likely to suffer from asthma and AR. In general, allergic diseases develop in a chronological order, beginning with AD and IgE-mediated FA in infancy, followed by AR and asthma in childhood. The natural progression of allergic manifestations is. The natural progression of allergic disease manifestations is defined as the “atopic march” (5). In industrialized societies, the prevalence of allergic diseases has increased dramatically in recent decades, affecting more than 30% of the world population (6). The growing prevalence of allergic diseases has placed a heavy medical and socioeconomic burden on the world (7). However, the precise mechanisms contributing to the development of allergic diseases remain largely unknown.

Notably, the growing global allergy epidemic is closely linked to drastic lifestyle and environmental changes, such as progressive urbanization and industrialization (8). These changes alter the function and composition of the human microbiota that primarily colonizes the gastrointestinal tract, skin, and airway (9). Host-microbiota interactions are vital in promoting health or causing disease development and progression (10, 11). In fact, aberrations in the communication between the host and the microbiota are considered to negatively affect immune homeostasis, contributing to immune hypersensitivity to allergens (12). Recent microbiome studies have highlighted the central role of the host microbiota in establishing an immunological equilibrium that shapes protection from or development of allergic diseases (13, 14). On a clinical level, this association can be traced to observations made in the 1980s that children in larger families were less likely to suffer from AD and AR than those in smaller families (15). The hygiene hypothesis was derived from these observations. Subsequent epidemiologic research yielded data showing that increased early-life microbial exposure (e.g., exclusive breast-feeding, prebiotic or probiotic use, absence of early antibiotic exposure, vaginal delivery, furry pet exposure during childhood, and farm life) is linked to a lower incidence of allergic diseases (16–19). Taken together, this clinical evidence has created a strong connection between the development of allergic disease and microbiota.

With the rapid development of microbiomics, numerous studies have been performed on the association between the microbiota and allergic diseases. It remains challenging, however, to comprehensively assess the research status of this area. Bibliometrics provides researchers with an objective and efficient means to evaluate the productivity, quality, impact, structure, interconnectivity, and progress of scientific publications by utilizing statistical and quantitative approaches (20). Unlike traditional literature reviews and meta-analyses, bibliometric analysis enables visual representation of scientific knowledge and identification of research hotspots and trends in a specific field, thus providing scholars with a diverse perspective on this field (21). Bibliometrics has become a highly valued tool for analyzing scientific research in the field of medicine worldwide over the past decade (22). There have been previous bibliometric studies that examined the microbiota in gastric cancer (23), the gut microbiota in cardiovascular diseases (24, 25), and the intestinal microbiota in depression (26). However, no bibliometric studies have focused on the microbiota in allergic diseases. Therefore, we aimed to analyze the general features, research hotspots, evolution routes, and emerging trends of the association between allergic diseases and the microbiota using bibliometric approaches. This research will assist in filling research gaps, provide greater insight into the latest perspectives on the microbiota in allergic diseases, and ultimately move the field forward.

## Materials and methods

### Data collection

**Figure 1:**
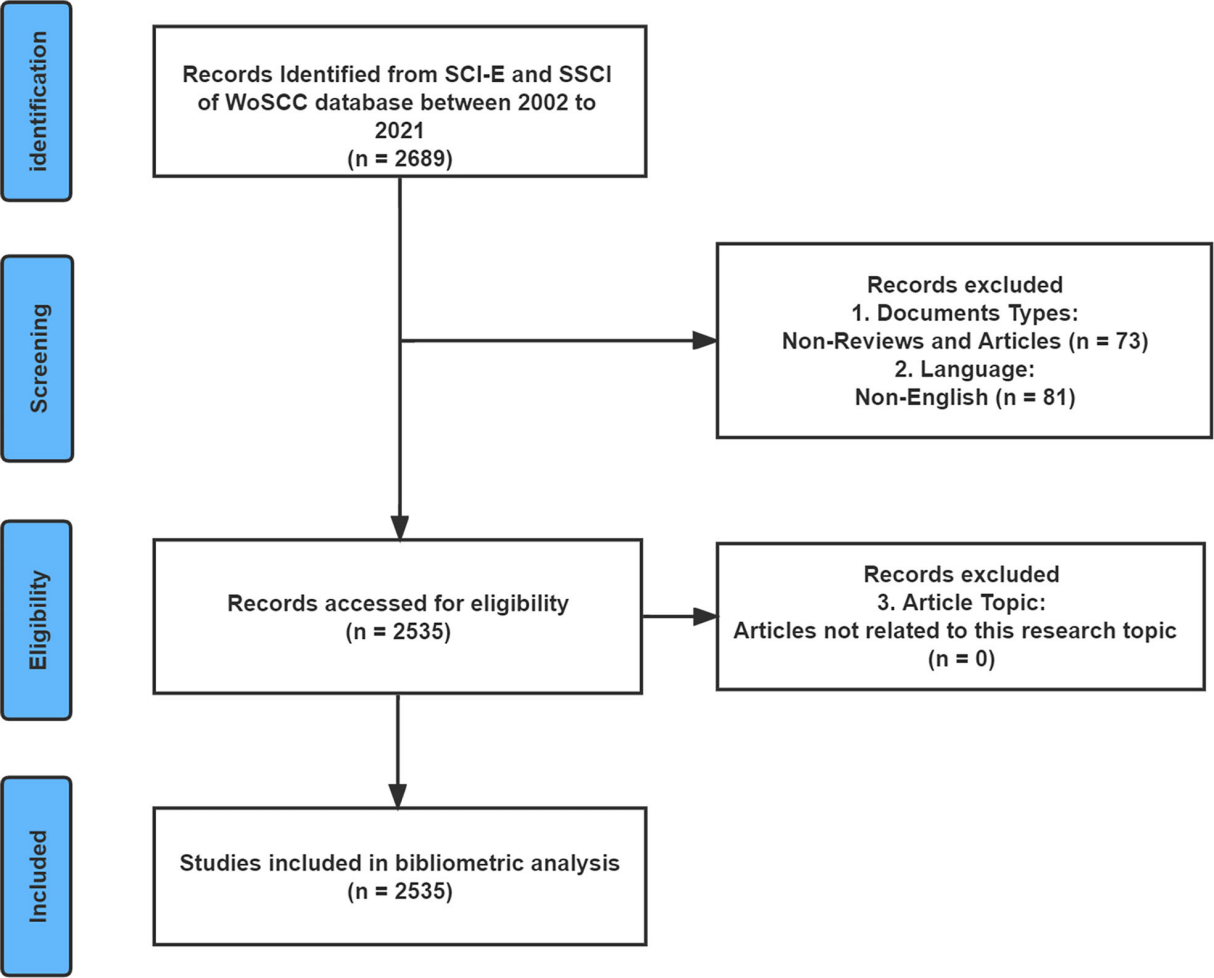
The flow diagram of literature enrollment and data screening.

The Web of Science Core Collection (WoSCC) is the world’s most influential citation-based database covering a comprehensive range of research types and is used extensively for bibliometric analysis (20, 21). Therefore, the data used in this study were downloaded from the Science Citation Index-Expanded (SCI-E) and Social Sciences Citation Index (SSCI) of the WoSCC. To avoid bias in data updates, the search was completed on 15 August 2022. The search strategy was [(allergic disease*) OR (atopic disease*) OR (allergy*)] AND [(microbiome*) OR (microbiota*) OR (microflora*) OR (flora*) OR (microorganism*) OR (microecology*)], and the publication years were limited to the period from 2002 to 2021. The language of the publications was “English”. “Articles” and “reviews” were the only publication types available. **Figure 1** depicts the flow chart of literature search and screening for this study. After completing the primary data collection, two investigators (HL and YW) excluded literature that did not fit the theme of this study. Divergent viewpoints between the two researchers’ results were resolved through discussions or consultation with experts (QH and YX) in the field.

### Data analysis

The bibliographic records were exported and saved in the form of “Full Record and Cited References” and “Plain Text”. Eventually, these data were imported into VOSviewer and CiteSpace for further analysis. In addition, we obtained the quartile in category and impact factor (IF) of journals from Journal Citation Reports (JCR) 2021.

CiteSpace is Java-based bibliometric software that can be used to discover knowledge structures, research hotspots, critical points, and future trends in a scientific field (22). There were three standardized algorithms in CiteSpace: the Jaccard similarity algorithm, the Cosine similarity algorithm, and the Dice similarity coefficient. The log-likelihood ratio (LLR) algorithm was used to performed CiteSpace clustering analysis. Moreover, an algorithm developed by Kleinberg (27), called burst detection, was applied to CiteSpace in order to detect rapid changes in the popularity of keywords or references over a given period of time. In this paper, we applied CiteSpace to perform collaboration network analysis of institutions, create dual-maps of journals, detect keywords and references with citation bursts, conduct cocitation analysis of references, and produce a timeline view of keywords and cocitation references. The specific settings used in CiteSpace were established as follows: time slicing: 2002-2021, years per slice: 1 year. Other parameters including term source, link strength and scope, and selection criteria were set to the default settings. The Pathfinder algorithm was employed to simplify the network structure and emphasize key features of the network in this paper.

VOSviewer, science mapping software, can construct and illustrate bibliometric network maps of countries/regions, journals, authors, or keywords according to data on bibliographic coupling, coauthorship relations, citations, and cocitations (24). The co-occurrence matrix is used as the basis for this software, and its clustering algorithm is dependent on the strength of association. With the help of the software’s distance visualization function, the similarity between the nodes is displayed. In general, distances between nodes with greater similarity are closer, while distances between nodes with smaller similarity are further. It is particularly useful for visualizing large-scale data intuitively (25). In the present study, this tool was used to produce visualization networks, including those based on keyword co-occurrence and coauthorship. In the VOSviewer software parameter setting, “full counting” was selected for the counting method, indicating each co-occurrence or co-citation link could have the same weight. In coauthorship analysis, the minimum number of documents per author was set to five. A threshold of twenty occurrences was set for the minimum number of occurrences in the analysis of keyword co-occurrence.

Microsoft Excel 2019 was used to present the volume of publications each year. Additionally, the online bibliometric platform (https://bibliometric.com) was used to analyze the collaboration network and publication output of countries.

## Results

### Analysis of publication trends

**Figure 2:**
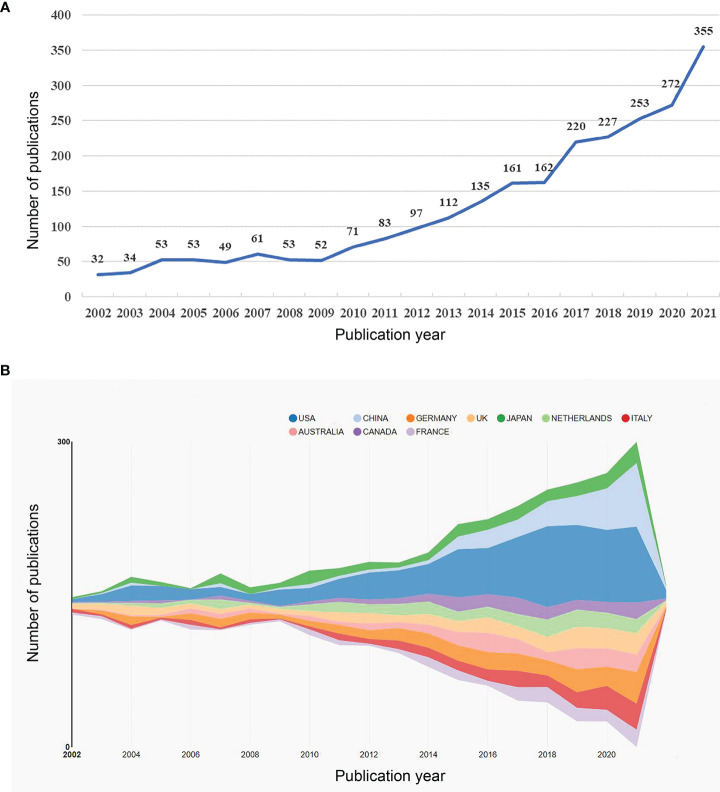
**(A)** The annual number of publications related to microbiota in allergic diseases (2002–2021). **(B)** Top 10 productive countries related to microbiota in allergic diseases (2002–2021).

A total of 2535 papers meeting the inclusion and exclusion criteria were published between 2002 and 2021. **Figure 2A** shows an overall increasing trend in the number of publications, despite fluctuating declines at certain points in time. Notably, the global interest in the role of the microbiota in allergic diseases peaked in 2017-2021, with 1327 publications in those five years, which accounted for more than 50% of the total publications.

### Contributions of countries/regions and institutions

**Figure 3:**
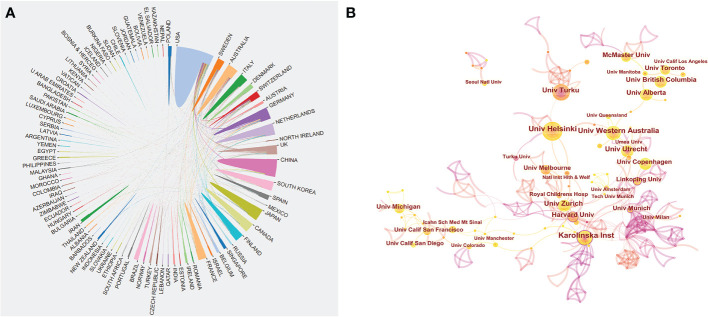
**(A)** Cooperation network of prolific countries/regions. **(B)** Visualization map of institutions’ cooperative relations.

A total of 94 countries/regions and 2845 institutions produced these publications. As shown in **Table 1** and **Figure 2B**, the USA was the most productive country (694, 27.3%), followed by Germany (244, 9.6%), China (204, 8.0%), the UK (189, 7.5%), and Japan (183, 7.2%). The centrality score is an important indicator to quantitatively assess the significance of nodes in a network (26). More frequent cooperation is associated with greater centrality in a collaborative network. **Figure 3A** shows cooperation networks across countries/regions. The top five countries/regions ranked by their centrality were the USA (0.71), the UK (0.20), Canada (0.14), the Netherlands (0.13), and Japan (0.07). Accordingly, the USA was determined to be the most influential country in the field based on publications and centrality.

**Table 1:** Ranking of top-10 countries that have published the most article from 2002 to 2021.

Rank	Article counts	Centrality score	Country
1	694	0.71	USA
2	244	0.00	Germany
3	204	0.03	China
4	189	0.20	UK
5	183	0.07	Japan
6	182	0.13	Netherlands
7	179	0.06	Italy
8	166	0.01	Australia
9	138	0.14	Canada
10	134	0.03	France

**Table 2** illustrates the top 10 most productive institutions. The University of California System published 84 papers and was the institution with the greatest contribution to this area, followed by UDICE-French Research Universities (72), the Karolinska Institute (70), Utrecht University (69), and the University of Helsinki (66). The cooperation network map according to institutions is shown in **Figure 3B**. We could see that many institutions cooperated actively, such as the Karolinska Institute, the University of Western Australia, the University of Helsinki, and the University of Turku. Notably, the top 10 institutions are all from economically developed countries such as those in Europe, the USA and Australia.

**Table 2:** Ranking of top-10 institutions from 2002 to 2021.

Rank	Article count	Institution	Country	Centrality score
1	84	University of California System	USA	0.01
2	72	UDICE-French Research Universities	France	0.00
3	70	Karolinska Institute	Sweden	0.20
4	69	Utrecht University	Netherlands	0.03
5	66	University of Helsinki	Finland	0.07
6	65	Harvard University	USA	0.11
7	58	University of Zurich	Switzerland	0.05
8	56	University of London	UK	0.00
9	54	University of Turku	Finland	0.05
10	51	University of Western Australia	Australia	0.08

### Authors and co-cited authors

**Figure 4:**
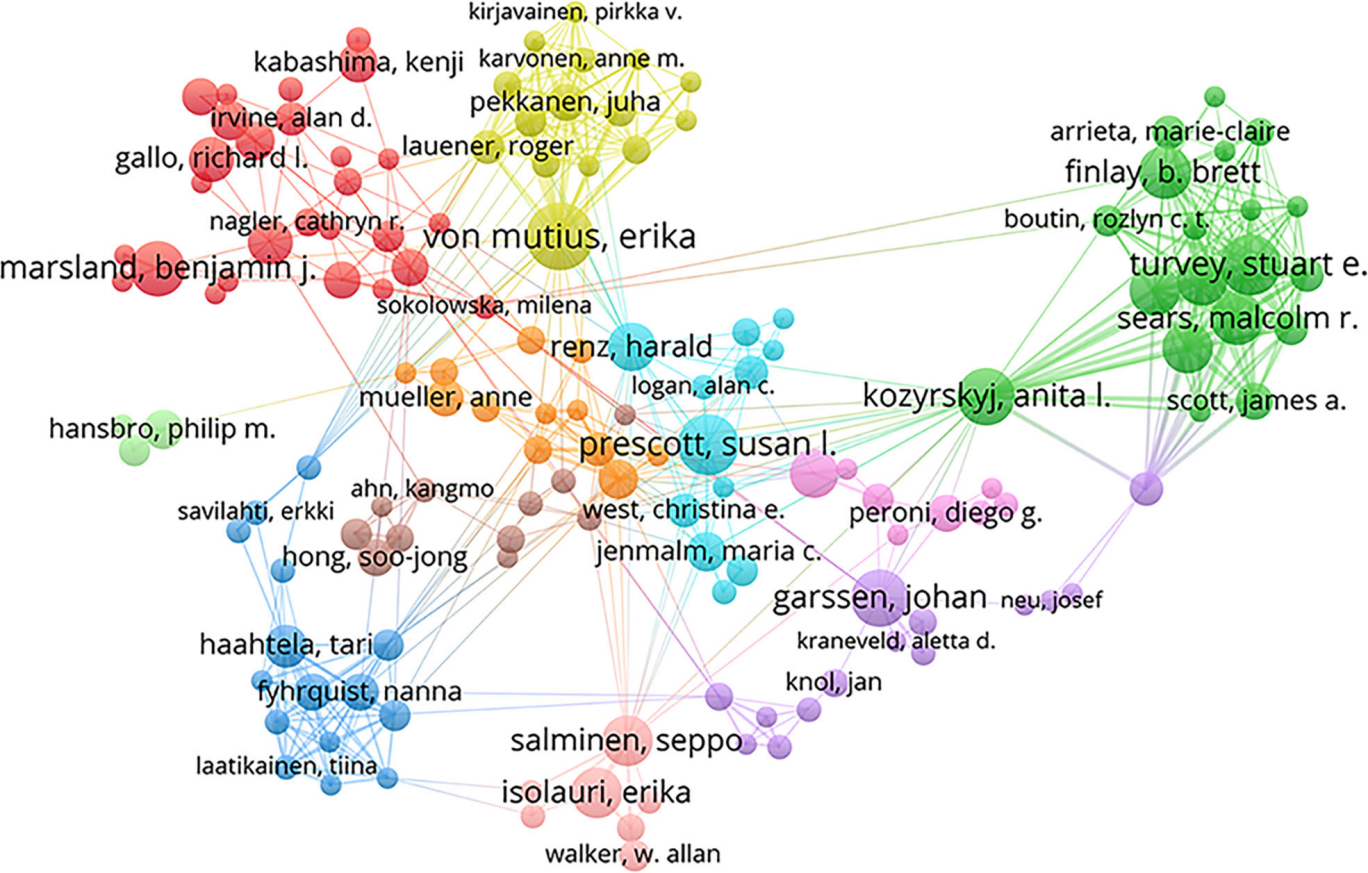
Collaborative network map among authors.

In total, 11457 authors contributed to this area. ​**Table 3** shows the top 10 most productive scholars. Isolauri E of the University of Turku was the most prolific scholar, with 37 publications, followed by Salminen S of the University of Turku (26), Kozyrskyj AL of the University of Alberta (22), Turvey SE of the University of British Columbia (21), and Sears MR of McMaster University (20). According to total citations, Isolauri E was ranked first with 3596 citations, followed by Marsland BJ (3200) and Salminen S (3052). The uppermost H-index value was that for Isolauri E. The results indicated that these authors have made great contributions to the field, especially Isolauri E. In this field, several academic teams have emerged as dominant players, as shown in **Figure 4**. There were usually close collaborations within the same cluster, such as that between Isolauri E and Salminen S and that between Kozyrskyj AL and Turvey SE. In addition, we observed that there was active cooperation among the clusters, such as for Isolauri E and Laatikainen T, Kozyrskyj AL and Peroni DG, and Marsland BJ and von Mutius E.

**Table 3:** Ranking of top-10 most productive authors from 2002 to 2021.

Rank	Author	Article count	Centrality score	Total number of citations	Average number of citations	H-index
1	Isolauri E	36	0.00	3596	99.89	26
2	Salminen S	26	0.00	3052	117.38	22
3	Kozyrskyj AL	22	0.07	1610	73.18	17
4	Turvey SE	21	0.00	1715	81.67	12
5	Sears MR	20	0.00	2155	107.75	13
6	Haahtela T	19	0.00	2168	114.11	14
7	Becker AB	19	0.03	1922	101.16	12
8	Marsland BJ	18	0.00	3200	177.78	14
9	Jenmalm MC	18	0.00	2697	149.83	17
10	Tang M	18	0.00	1353	75.17	16

### Analysis of journals

Academic journals serve as a key medium for presenting the results of scientific research. The 2535 included publications were published in 741 academic journals. **Table 4** presents the characteristics of the top 10 most productive journals. There were 563 publications in the top 10 most productive journals, which accounted for 22.2% of the total articles published. Most of these journals specialize in the field of allergy and immunology. In terms of the number of publications, the most influential journal in this field was the *Journal of Allergy and Clinical Immunology* (128), followed by *Clinical and Experimental Allergy* (77) and *Frontiers in Immunology* (67). Six of the top 10 journals were located in the first quartile (Q1), and eight had an IF above 5.0. According to the cocitation count, the top three journals were the *Journal of Allergy and Clinical Immunology* (11717), *Clinical and Experimental Allergy* (6344), and *Allergy* (2971).

**Table 4:** Ranking of top-10 most productive journals from 2002 to 2021.

Rank	Journal	Article count	Country	Journal citation reports (2021)	Impact factor (2021)	Total number of citations	Mean number of citations	H-index
1	*Journal of Allergy and Clinical Immunology*	128	USA	Q1	14.29	11717	96.83	65
2	*Clinical and Experimental Allergy*	77	UK	Q2	5.401	6344	82.39	41
3	*Frontiers in Immunology*	67	Switzerland	Q1	8.786	1424	21.25	22
4	*Allergy*	64	UK	Q1	14.71	2971	46.42	31
5	*Plos One*	49	USA	Q2	3.752	2034	41.51	22
6	*Pediatric Allergy and Immunology*	46	Denmark	Q1	5.464	1995	43.37	25
7	*Current Opinion in Allergy and Clinical Immunology*	39	USA	Q3	3.253	1341	34.38	20
8	*Nutrients*	37	Switzerland	Q1	6.706	876	23.68	19
9	*International Journal of Molecular Sciences*	33	Switzerland	Q1	6.208	800	24.24	13
10	*Annals of Allergy Asthma & Immunology*	24	USA	Q2	6.248	527	21.96	13

**Figure 5:**
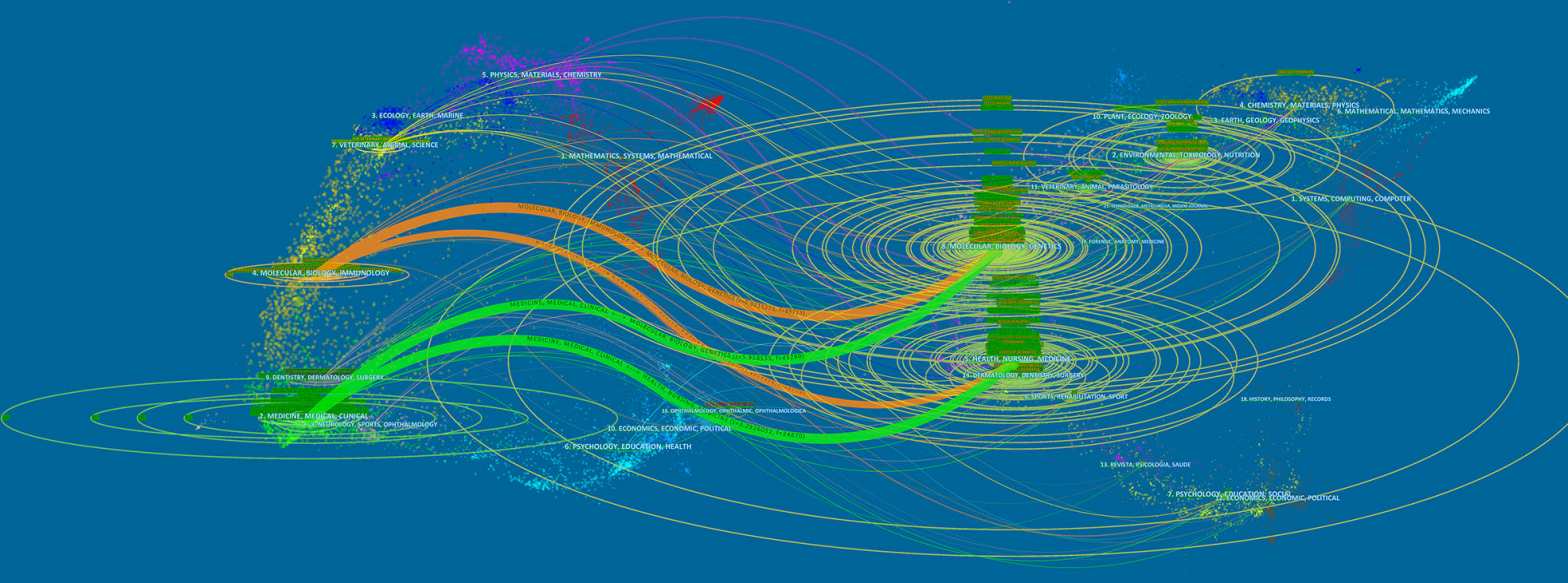
A dual-map overlay analysis of journals.

The dual-map overlay of journals shows the topic distribution of relationships between journals. On the left side of the map are the citing journals, while on the right side are the cited journals. There are four primary citation paths in **Figure 5**. The two green routes indicate that the papers published in molecular/biology/genetics journals were generally cited by medicine/medical/clinical journals and that the studies published in health/nursing/medicine journals were generally cited by medicine/medical/clinical journals. As the two orange paths show, the articles published in molecular/biology/immunology journals often cited molecular/biology/genetics journals and health/nursing/medicine journals. Overall, this field mainly involves subdisciplines in medicine, molecular biology, and immunology.

### Analysis of keyword co-occurrence and bursts

**Figure 6:**
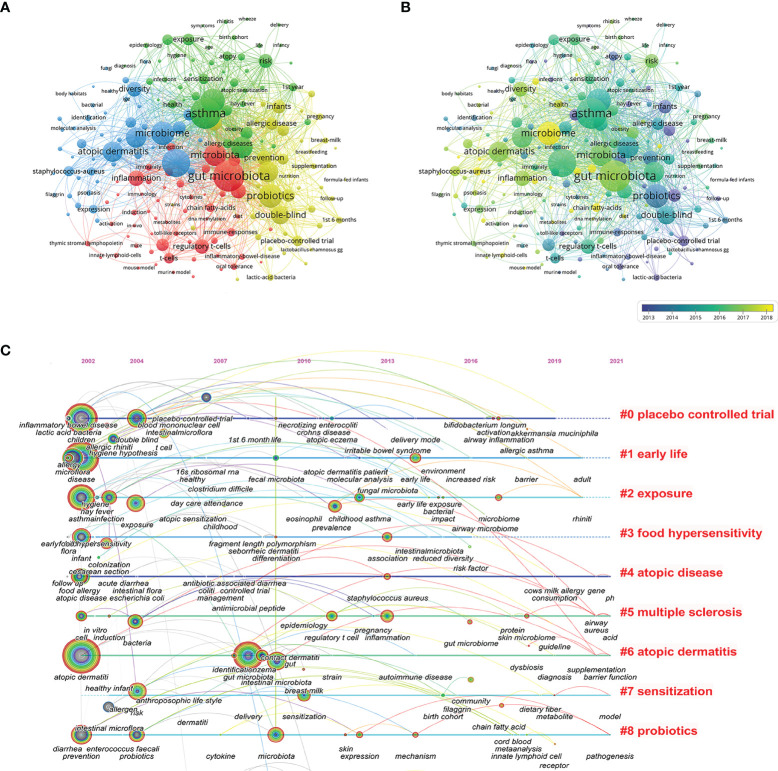
Keywords analysis for research of microbiota in the allergic diseases field (2002–2021). **(A)** Clustering co-occurrence map of keywords. **(B)** Distribution of keywords based on the average time of appearance. **(C)** Time-zone view of keyword co-occurrence.

Analysis of co-occurring keywords can be used to identify research hotspots within a certain knowledge domain. A total of 6896 keywords were extracted, of which 199 keywords occurred more than 20 times. **Figure 6A** presents the keyword co-occurrence visualization map, in which the occurrence of the keyword indicates the size of each node. The top 5 keywords with the highest co-occurrence frequency were “gut microbiota” (459), “asthma” (449), “atopic dermatitis” (401), “children” (322), and “probiotics” (319). Cluster analysis of keywords can be used to determine the structural system of a related research field. All keywords could be clustered into four groups represented by different colored circles based on the link strength of the keyword co-occurrence (**Figure 6A**). The green cluster mainly represents respiratory allergies. The red cluster is primarily related to immunological and molecular mechanisms. The yellow cluster is focused on probiotics and breast-feeding. The blue cluster 4 represents the microbiome and atopic dermatitis. The color depth in the area demonstrates that keywords were distributed in chronological order (**Figure 6B**). Before 2012, most research concentrated on “atopy”, “infants”, and “placebo-controlled trial”, while the latest identified research hotspots indicated “microbiome”, “chain fatty acids”, and “innate lymphoid cells” as emerging fields. Additionally, a time-zone view of keyword co-occurrence was produced (**Figure 6C**), which assisted in visualizing the phased research hotspots and directions from the temporal dimension.

**Figure 7:**
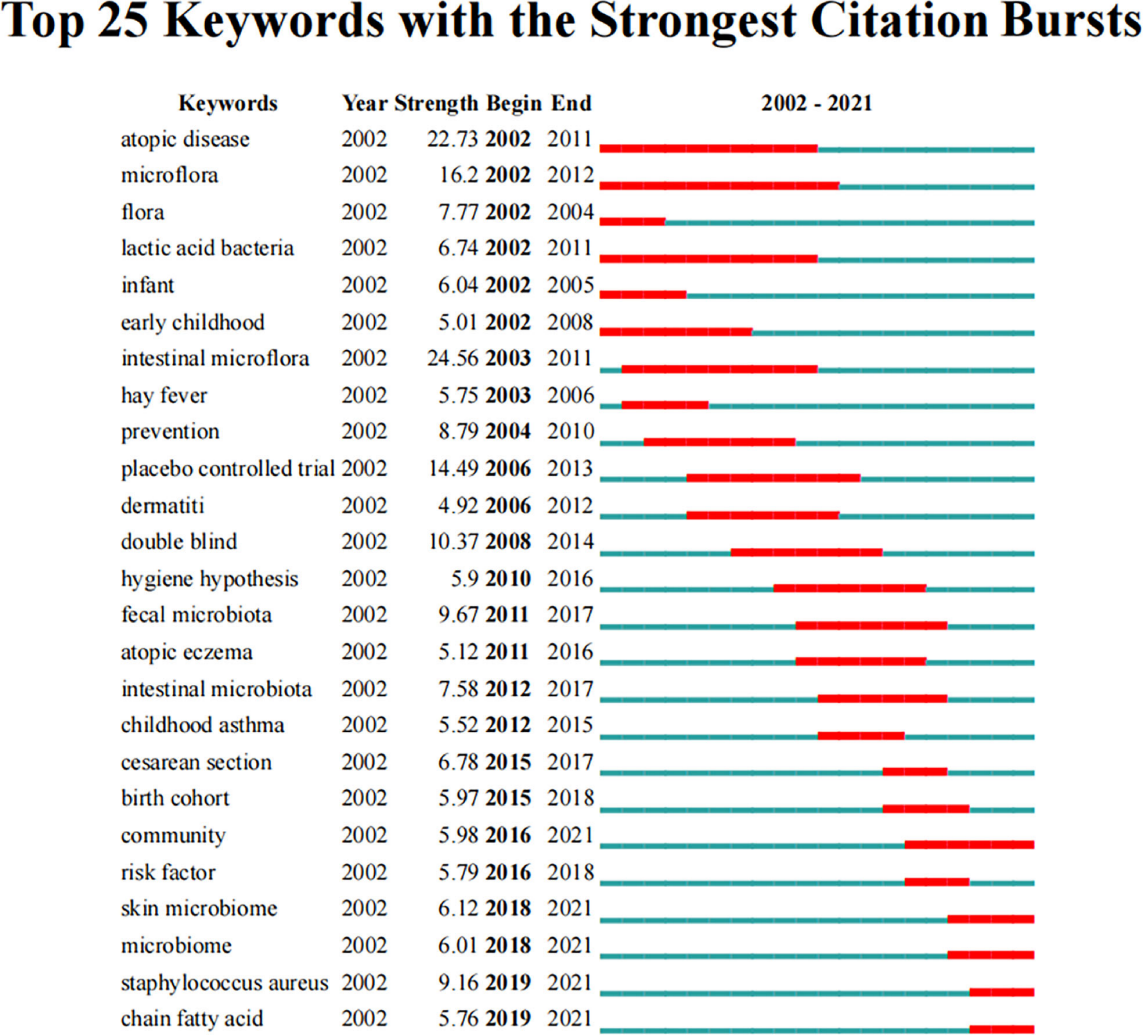
Top 25 keywords with the strongest citation bursts related to microbiota in allergic diseases (2002–2021).

Keyword bursts can elucidate the development trends of the field. **Figure 7** shows the top 25 keywords with the strongest citation bursts. From 2002 to 2021, “intestinal microflora” had the highest burst strength (24.56), followed by “atopic disease” (22.73) and “placebo-controlled trial” (14.49). Moreover, “community”, “skin microbiome”, “microbiome”, “*Staphylococcus aureus*”, and “chain fatty acid” had bursts that lasted until 2021, which reflect the latest research trends.

### Analysis of co-cited references and reference bursts

Co-cited references refer to those that are cited by one or more publications at the same time that represent the knowledge bases of a particular area (25). **Table 5** lists the top 10 co-cited articles. Among them, nine papers were co-cited over 300 times. The paper published by Brown et al. (28) in *Science Translational Medicine* (2292) is the most highly cited, followed by those by Atarashi et al. (29) in *Nature* (1665) and Trompette et al. (30) in *Nature Medicine* (1448).

**Table 5:** Top-10 most co-cited references from 2002 to 2021.

Rank	Title	Author	Year	Journal	Citation frequency
1	Hidden Killers: Human Fungal Infections	Brown GD	2012	*Science Translational Medicine*	2292
2	T-reg induction by a rationally selected mixture of Clostridia strains from the human microbiota	Atarashi K	2013	*Nature*	1665
3	Gut microbiota metabolism of dietary fiber influences allergic airway disease and hematopoiesis	Trompette A	2014	*Nature Medicine*	1448
4	Prebiotic effects: metabolic and health benefits	Roberfroid M	2010	*British Journal of Nutrition*	1279
5	Microbial Exposure During Early Life Has Persistent Effects on Natural Killer T Cell Function	Olszak T	2012	*Science*	1036
6	Exposure to Environmental Microorganisms and Childhood Asthma	Ege MJ	2011	*New England Journal of Medicine*	990
7	Temporal shifts in the skin microbiome associated with disease flares and treatment in children with atopic dermatitis	Kong HDH	2012	*Genome Research*	951
8	Bioaerosol health effects and exposure assessment: Progress and prospects	Douwes J	2003	*Annals of Occupational Hygiene*	874
9	Early infancy microbial and metabolic alterations affect risk of childhood asthma	Arrieta MC	2015	*Science Translational Medicine*	847
10	The human skin microbiome	Byrd AL	2018	*Nature Reviews Microbiology*	675

**Figure 8:**
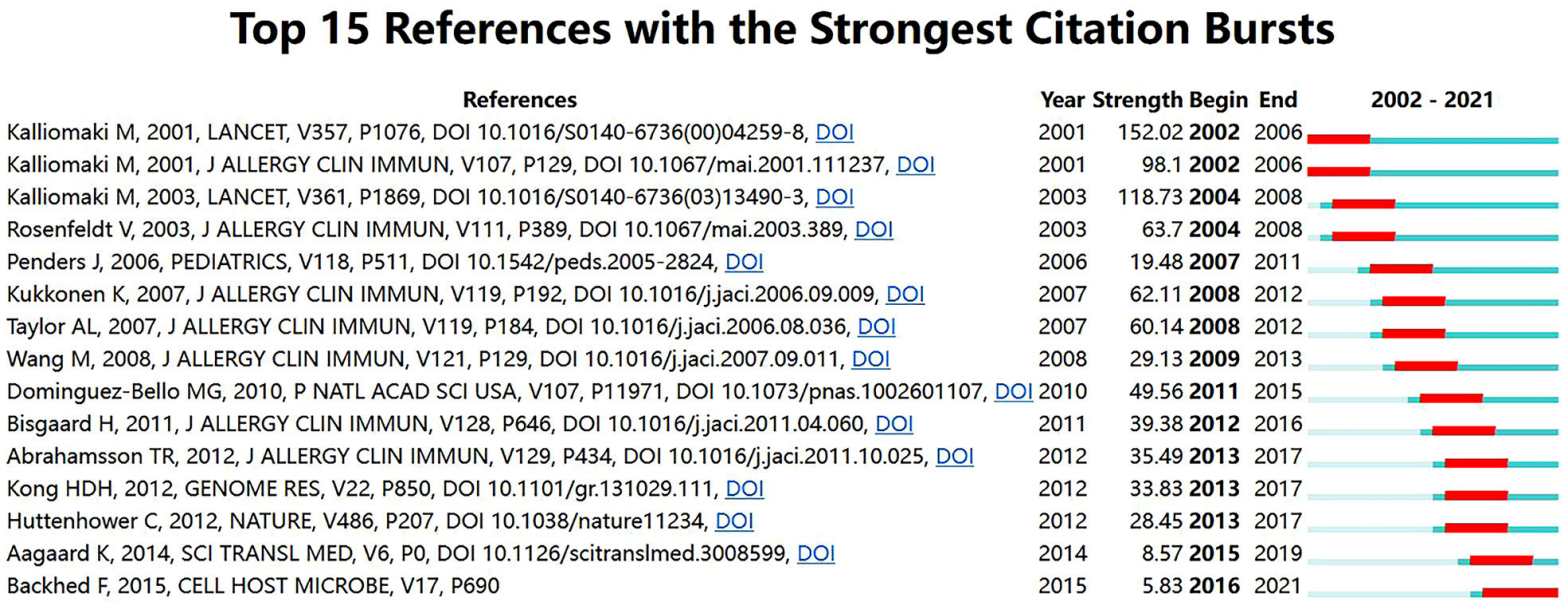
Top 15 references with the strongest citation bursts related to microbiota research in the allergic diseases field (2002–2021).

Detection of references with citation bursts can indicate the evolution of hotspots over time and future trends in a certain field (26). **Figure 8** displays the top 15 references with the strongest citation bursts. The article with the strongest burst (28.43), entitled “Probiotics in primary prevention of atopic disease: A randomized placebo-controlled trial”, was published in *Lancet* by Kalliomaki et al. (31) in 2001. Additionally, the paper entitled “Dynamics and Stabilization of the Human Gut Microbiome during the First Year of Life” (32) had a burst that lasted until 2021.

**Figure 9:**
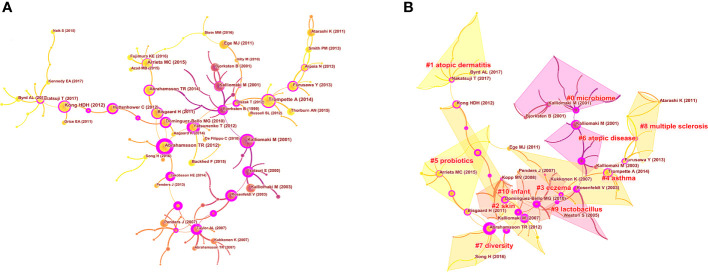
Analysis of co-cited references in the field of microbiota in allergic diseases (2002–2021) **(A)** The network of co-cited references. **(B)** Clustering visualization map of the co-cited references.

**Figure 10:**
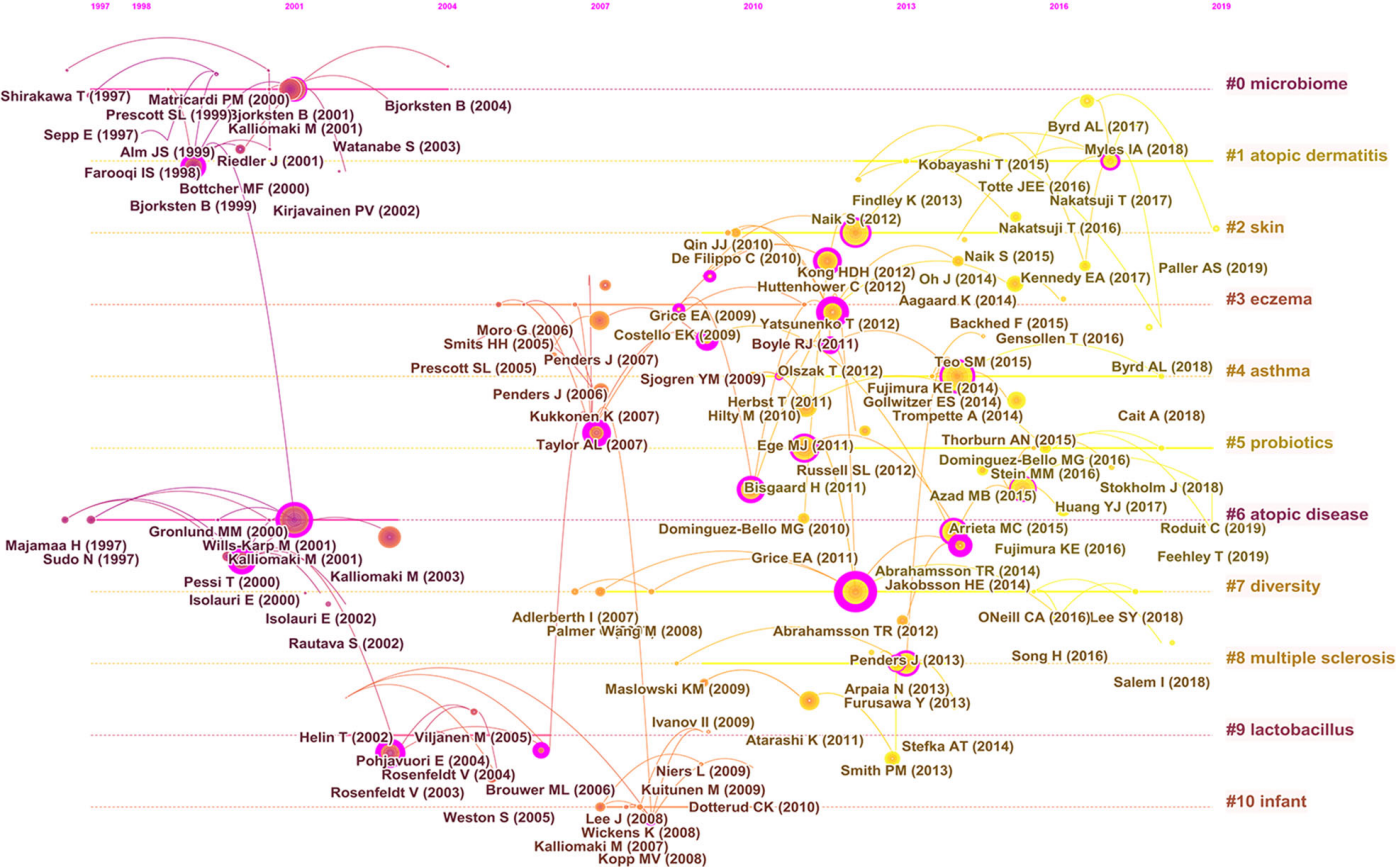
Timeline visualization map of the co-cited references.

As shown in **Figure 9A**, the cited reference network consisted of 122 nodes and 124 links that represented the cocitation relationships between the references. There are links between nodes showing the frequency with which the same article was cited. The diameter of a node is proportional to the frequency with which the cited reference is quoted. **Figure 9B** illustrates the largest 11 clusters of the reference cocitation network, which represent the 11 main research topics in the field. The five largest clusters included ‘‘microbiome’’ (#0), ‘‘atopic dermatitis’’ (#1), ‘‘skin’’ (#2), “eczema” (#3), and “asthma” (#4). An illustration of the scientific relevance of cocited references over time is shown in **Figure 10**, which was produced by plotting the evolution of the nine largest clusters on a timeline. The most recent were “atopic dermatitis’’ (#1), “skin” (#2), “asthma” (#4), “probiotics” (#5), and “atopic diseases” (#6).

## Discussion

Over the past few decades, the prevalence of allergic diseases has risen dramatically and has become a major global health problem. Numerous studies have revealed the central role of microbiota dysbiosis in the development or progression of allergic diseases. Especially in the last 20 years, there have been extensive efforts by researchers to clarify the connection. With the benefit of bibliometrics, we can gain a deeper understanding of the research status in a particular field as well as anticipate future trends. To the best of our knowledge, this is the first study to examine the association between the microbiota and allergic diseases based on bibliometric and visual analysis.

### General information

According to the WoSCC database, 11457 authors from 2845 institutions in 94 countries/regions published 2535 papers in 741 academic journals from 2002 to 2021. In the last two decades, a significant increase has been observed in the output of publications regarding the association between the microbiota and allergic diseases. The overall increasing trend of publication also indicates that this field is a hot topic and will continue to attract increased attention in the future.

Visual analysis of contributions by country/region showed that the USA has a distinct advantage in research on the microbiota and allergic diseases. The number of papers published in the USA represented 27.3% of the total publications. In addition, the USA had a maximum centrality of 0.71, indicating that they played a central role in fostering international cooperation. Notably, China is the only developing country in the top 10, with 204 publications in this area. This may be partly due to the key research and development project on biomacromolecules and the microbiome issued by the Ministry of Science and Technology of China. In general, economic imbalances and differences in policy specifications among countries might contribute to regional imbalances in knowledge production on the microbiota and allergic diseases.

Nearly 3000 institutions worldwide have produced studies on the microbiota in allergic diseases. The University of California System was the most productive institution. As shown in **Figure 3B**, while many institutions collaborated actively, some did not. Therefore, we strongly recommend that countries and institutions involved in similar research topics enhance cooperation and work together to develop and expand this field.

Among the numerous researchers, Isolauri E, Salminen S, Kozyrskyj AL, Turvey SE and Sears MR have made the greatest contributions to this field, which can be attributed to their large number of publications. Remarkably, Isolauri E, who is the head of the Department of Clinical Medicine, University of Turku, published the most papers and had the highest number of citations in this field. She has expertise in several areas, such as the gut microbiota, mucosal immunology, and probiotics. In terms of author cooperation networks, the field has been characterized by the establishment of numerous academic teams as well as close collaboration between different research communities. This situation has accelerated the rapid growth of the field to some extent.

It may be appropriate to select the top 10 journals that scholars read and publish their relevant papers in, as they have published a large number of articles in this area. Among the top 10 journals, six are located in the JCR Q1 zone, and 2 journals have an IF over 10, which indicated that the literature in this field is of relatively high quality. We also found that the top 10 journals published more than one-fifth (22.2%) of the total number of papers on microbiota research in the allergic disease field. The secondary disciplines of these journals mainly involve allergy and immunology. These results indicate that the current studies in the field of the microbiota and allergic diseases are relatively concentrated and focus on allergy and immunology.

### Emerging topics

The evolution of references with citation bursts partially reveals the dynamic changes in a specific area (25). The strongest citation burst was from an article in *Lancet* published by Kalliomaki et al. (31) in 2001 (152.02, 2002-2006). This randomized placebo-controlled trial explored for the first time the role of probiotics in the primary prevention of atopic diseases. Specifically, their results showed that *Lactobacillus* GG was an efficacious treatment for preventing the development of atopic disease in children at high risk. More importantly, this work proposed that the gut microbiota may act as an important source of probiotics and natural immunomodulators that can prevent atopic diseases. In addition, a study by Bisgaard et al. (33) published in the *Journal of Allergy and Clinical Immunology* exhibited the highest citation burst in the last decade. This prospective cohort study implied that an increased risk of allergic disease at school age was linked to reduced diversity of the gut microbiota during infancy. Notably, the most recent citation burst occurred in 2016 and continues through 2021, which was for an article published by Bäckhed et al. (32) in *Cell Host Microbe* in 2015. This was a large metagenomic analysis of gut microbial genes during the first year of life, thus laying the groundwork for understanding the interaction between the human body and the gut microbiome in early life.

The research frontiers in a certain field can be captured by keywords with citation bursts. Notably, some keywords, such as “*Staphylococcus aureus*”, “skin microbiome”, “microbiome”, and “chain fatty acid”, currently have ongoing bursts, reflecting the latest research trends.

*Staphylococcus aureus* (*S. aureus*) is a frequent colonizing microbe in humans (34). However, a growing number of studies have reported a central role for *S. aureus* in allergic diseases. Over 90% of all patients with AD, regardless of the timing of presentation, have *S. aureus* colonization (35). During an AD flare, there is a decline in the skin microbiome diversity, with *S. aureus* becoming dominant. Moreover, the increased proportion of *S. aureus* relative to other commensals was linked to more severe AD and barrier dysfunction (36, 37). Mechanistic studies have revealed that *S. aureus* influences AD in multiple ways. Many *S. aureus* proteins, such as α-toxin and serine protease, are capable of causing membrane damage/lysis of keratinocytes, thereby triggering epidermal barrier dysfunction (38). *S. aureus* can also directly trigger mast cell degranulation and promote the overproduction of type-2 cytokines through its superantigens, such as enterotoxins and toxic shock syndrome toxin (TSST) (38). Moreover, in response to *S. aureus* invasion, keratinocytes release alarmins such as TSLP, IL-25, and IL-33, further amplifying a Th2 response (37). A recent study showed that epicutaneous *S. aureus* could stimulate the release of IL-36 from keratinocytes, which directly triggered IgE class switching in B cells and increased IgE production (39). Additionally, *S. aureus* contributes to persistent Th2 airway inflammation in asthma. Kim et al. (40) reported a significant association between the prevalence and activity of asthma and nasal *S. aureus* colonization. A recent cohort study showed that staphylococcal enterotoxin B (SEB)-IgE sensitization was an independent risk factor for severe asthma (41). Further study has shown that the IL-33/ST2 pathway is critical in *S. aureus*-induced type-2 airway inflammation (42). In addition, disturbance of the mucosal microbiota of the inferior turbinate, especially due to an increase in *S. aureus* abundance, is associated with high levels of total IgE in AR (43). New findings suggest that *S. aureus*-dominant dysbiosis occurs in the microbiota of the nasal mucus from patients with AR (44). Serological evidence of formation of IgE against superantigens derived from *S. aureus* has also been identified in people with AR (45). Compared to healthy subjects, AR patients had higher levels of IgE against SEA, SEB and TSST.

The microbiome refers to the sum of the microorganisms, their collective genomes and environmental interactions in a specific ecological niche (9). The term is often used interchangeably with microbiota (46). As mentioned earlier, the rising prevalence of allergic diseases is attributed in large part to the perturbation of the human microbiome (11). The rapid advances in next-generation sequencing technologies, omics analysis, and computational biology have greatly advanced our understanding and characterization of the human microbiome (47). Despite the dramatic increase in microbiome research, our knowledge of the microbiome, especially its role in health and disease, remains preliminary. It may be possible to develop a precision medicine approach by diving deeper into an individual’s unique microbiome. As an important component of the human microbiome, the skin microbiome is integral to maintaining skin homeostasis and regulating immune function. Notably, dysbiosis of the skin microbiome promotes progression through the atopic march and the development of allergic diseases (37).

Recently, scholars have focused their attention on the role of gut microbiota-derived metabolites, particularly fatty acids, in allergic diseases. Short-chain fatty acids (SCFAs), including butyrate, acetate, and propionate, are the major dietary fiber metabolites produced by the intestinal microbiota and have several antiallergic properties (48). SCFAs are effective in protecting against food allergy (48). Children with the highest level of butyrate were less likely to have atopic sensitization and asthma (49), and oral administration of SCFAs attenuated allergic airway inflammation in murine studies (50). Mechanistically, SCFAs induce enhanced phagocytosis by dendritic cells but diminish their ability to promote Th2 cell effector function, which is mediated by G protein-coupled receptor 41 (30). SCFAs can also strengthen the function and induction of regulatory T (Treg) cells through epigenetic mechanisms, such as the enhanced acetylation of the Foxp3 promoter, leading to long-term antiallergic effects (51). Butyrate inhibits eosinophil survival and migration (52) as well as mast cell activation (53) by inducing histone acetylation. A recent study showed that SCFAs promote the maintenance of regulatory B (Breg) cell function by stabilizing IL-10 expression in Breg cells, thereby alleviating allergic inflammation (54). In addition, acetate and butyrate have a preventive or therapeutic effect on allergic diseases by enhancing the barrier function of the intestinal epithelium (48).

Long-chain fatty acids are important nutrients that serve not only as energy sources but also as immune system regulators. It has been shown that prenatal supplementation with omega-3 long-chain polyunsaturated fatty acids (LCPUFAs) can decrease the risk of allergic diseases in offspring (55). Breastmilk containing higher levels of ω-3 LCPUFAs was found to possibly lower the risk of sensitization at 2 years of age in a Swedish birth cohort (56). The proportions of ω-3 LCPUFAs in infant cord serum were negatively associated with allergies at the age of 3 or 8 years (57). In addition, a longitudinal study showed that a reduced risk of allergic diseases (aeroallergen sensitization, asthma, and rhinitis) at 16 years of age was linked to higher proportions of certain ω-3 LCPUFAs in plasma at 8 years of age (58). ω-3 LCPUFAs, such as eicosapentaenoic acid (EPA) and docosahexaenoic acid (DHA), exert antiallergic effects mainly through their conversion into lipid mediators. For instance, resolvin E1 (a metabolite of EPA) contributes to the resolution of airway inflammation in allergic asthma (59), and resolvin D1 (a metabolite of DHA) attenuates histamine responses in allergic conjunctivitis (60). Sawane et al. (61) reported that 15-hydroxyeicosapentaenoic acid (15-HEPE), an EPA-derived metabolite, could inhibit mast cell degranulation in a PPARγ-dependent manner, thereby improving allergic symptoms in mice with AR. It has been recently shown that maternal ω-3 LCPUFAs can ameliorate infant AD by increasing TRAIL expression in dermal plasmacytoid dendritic cells in mice (62).

Therefore, SCFAs and ω-3 LCPUFAs could be beneficial in the prevention or treatment of allergic disorders. However, the precise mechanisms need to be further investigated to facilitate clinical translation.

### Limitations

This study has several limitations inherent in bibliometrics. First, literature was extracted only from the WoSCC database, which may have led to incompleteness and bias in the included studies. Second, only English-language articles and reviews were included; therefore, our findings may not be comprehensive. Last, despite our standardized procedures, a certain bias should not be excluded since some keywords are phrased in different ways, some authors have the same name, the WoSCC database is continuously updated, etc. We believe that these limitations should be addressed wherever possible in the future. Despite these limitations, it can still be assumed that our study reflects the overall state and emerging trends in this area.

## Conclusion

The number of publications in this field has been rising rapidly over the past 20 years, indicating that scholars have focused on the role of the microbiota in allergic diseases. Despite the relatively close international cooperation among the institutes and authors in this field, there is still a need to strengthen academic exchange and cooperation. Several different topics have gradually emerged with the development of research in this field. Recent research focuses have included “atopic dermatitis’’, “skin”, “asthma”, “probiotics”, and “atopic disease”, which constitute an important research base in this field. “Community”, “skin microbiome”, “microbiome”, “*Staphylococcus aureus*”, and “chain fatty acid” were identified as the new research frontier in this field. Overall, this is the first bibliometric study that sheds an objective and systematic light on research on the association between the microbiota and allergic diseases, which will serve as a valuable guide for further study in this field.

## Data availability statement

The raw data supporting the conclusions of this article will be made available by the authors, without undue reservation.

## Author contributions

HL, YW, and YX designed the study. HL and YW collected the data. ZG, PL, and DQ analyzed the data. HL and YW wrote the manuscript. HL, YW, ZG, and QH prepared the figures and tables. QH and YX reviewed and revised the manuscript. All authors contributed to the article and approved the submitted version.

## Funding

This research was supported by the National Natural Science Foundation of China (No. 82071017), National Natural Science Foundation of Hubei Province (No. 2021CFB125) and the Fundamental Research Funds for the Central Universities (No. 2042021kf0093).

## Conflict of interest

The authors declare that the research was conducted in the absence of any commercial or financial relationships that could be construed as a potential conflict of interest.

## Publisher’s note

All claims expressed in this article are solely those of the authors and do not necessarily represent those of their affiliated organizations, or those of the publisher, the editors and the reviewers. Any product that may be evaluated in this article, or claim that may be made by its manufacturer, is not guaranteed or endorsed by the publisher.
